# Association between vitamin B1 intake and hyperuricemia in adults

**DOI:** 10.1038/s41598-024-66384-4

**Published:** 2024-07-16

**Authors:** Yi-Ming Li, Xiao-Hu Xu, Xiao-Fan Xu, Xia-Xia Yang, Yi-Long Dai, Dong-Xue Song, Cheng-Qiang Jin, Yan-Xia Jia

**Affiliations:** 1 College of Clinical Medicine, Jining Medical University, Jining, Shandong Province China; 2 Clinical Laboratory, Affiliated Hospital of Jining Medical University, Jining Medical University, Jining, Shandong Province China; 3 Radiology Department, Jining First People’s Hospital, Jining, Shandong Province China; 4 Clinical Laboratory Management Teaching and Research Office, College of Forensic Medicine and Medical Laboratory, Jining Medical University, Jining, Shandong Province China

**Keywords:** Hyperuricemia, Vitamin B1, NHANES, Prevalence, Uric acid, Diseases, Health occupations, Signs and symptoms

## Abstract

Studies investigating the relationship between dietary vitamin B1 intake and risk of Hyperuricemia (HU) are scarce, the present study aimed to examine the association of dietary vitamin B1 intake and HU among adults. This cross-sectional study included 5750 adults whose data derived from National Health and Nutrition Examination Survey (NHANES) from March 2017 to March 2020. The dietary intake of vitamin B1 was assessed using 24-h dietary recall interviews. The characteristics of study participants were grouped into five levels according to the levels of vitamin B1 quintile. Multivariate logistic regression analysis was used to estimate the odds ratio (OR) and 95% confidence interval (CI) of HU, according to the vitamin B1 intake quintile for male and female separately. The dose–response relationship was determined by the restricted cubic spline (RCS). Smoothed curve fitting was used to assess serum uric acid concentration versus dietary vitamin B1 intake in the study population. The prevalence of hyperuricemia was 18.90% (20.15% and 17.79% for males and females, respectively) in the United States from March 2017 to March 2020. Multiple logistic regression analyses showed that in the male population, the HU ratio (OR) of vitamin B1 intake in Q2 to Q5 compared with the lowest quintile (Q1) was 0.75 (95% CI 0.52, 1.09), 0.70 (95% CI 0.48, 1.02), 0.66 (95% CI 0.44, 0.99) and 0.55 (95% CI 0.34, 0.90). The *P* for trend was 0.028. In women, the ORs for vitamin B1 intake Q2 to Q5 were 0.87 (95% CI 0.64, 1.19), 0.97 (0.68–1.38), 1.05 (0.69–1.60) and 0.75 (0.42–1.34), respectively. The *P* for trend was 0.876. The RCS curve revealed a linear relationship between vitamin B1 intake and the risk of hyperuricemia in men (*P* nonlinear = 0.401). Smoothed curve fitting demonstrated a negative association between vitamin B1 intake and serum uric acid concentration in men, whereas there was no significant association between dietary vitamin B1 intake and the risk of hyperuricemia in women. In the US adult population, dietary vitamin B1 intake was negatively associated with hyperuricemia in males.

## Introduction

Uric acid is the final product of purine metabolism and the main endogenous antioxidant in the body. It plays bioprotective roles. Hyperuricemia (HU) occurs because of urate overproduction or impaired urate excretion through the kidney and gastrointestinal tract^1^. In recent decades, there is strong epidemiological evidence that the prevalence of hyperuricemia is on the increase worldwide^2^. HU is considered as a precursor of gout and an essential risk factor for cardiovascular diseases, type 2 diabetes, hypertension, and chronic kidney disease^3^. As HU receives more attention, it is necessary to find potentially modifiable factors that inhibit the increase in serum uric acid concentration.

Vitamin B1 or thiamin, is an essential water-soluble vitamin critical for carbohydrate and amino acid catabolism and gluconeogenesis. Vitamin B1 is essential for the proper function of most tissues and organs. Thus, its deficiency can have a myriad of clinical effects, most notably on the nervous and cardiovascular systems^4^. Dietary intake of vitamin B1 is closely related to human health. Studies over the past decade have focused on an association between thiamine deficiency and glucose metabolism dysfunction in patients with type 1 and type 2 diabetes. One of these mentioned that high doses of thiamine may improve the vascular complications of the disease, such as nephropathy, neuropathy and retinopathy^5^. Previous studies have found that HU may be associated with intake levels of multiple vitamins, such as vitamin C^1^, retinol^6^, vitamin B12^7^ and so on. However, studies investigating the relationship between dietary vitamin B1 intake and risk of HU are scarce, only one cross-sectional study has shown that dietary vitamin B1 intake is negatively associated with HU in men of China^8^.

To date, no known studies have explored the relationship between dietary vitamin B1 intake and HU. Therefore, the purpose of this cross-sectional study was to investigate this correlation using a large nationally representative sample in the US.

## Results

A total of 5750 individuals (2725 males, 3025 females) participated in our study. The characteristics of study participants were grouped into five levels according to the levels of vitamin B1 quintile, as shown in Table 1. Significant differences were detected across all quintiles of vitamin B1 intake for age, gender, race, educational background, BMI, HU prevalence, vitamin C intake, vitamin B6 intake, folate intake, retinol intake, energy intake, protein intake, carbohydrate intake, serum cholesterol, and TG levels. Subjects with higher levels of vitamin B1 intake were younger, more non-Hispanic whites, and more males than females, had higher intakes of vitamin C, dietary vitamin B6, folic acid, retinol, and energy, protein, and carbohydrate intakes, were also likely to have lower BMI, serum cholesterol, and TG, and were less likely to have hypertension, diabetes, and HU.

**Table 1:** Characteristics of the participants according to intake of vitamin B1.

Characteristics	Vitamin B1 intake (mg/Day)					*P*-value
	Q1 (n = 1150)	Q2 (n = 1149)	Q3 (n = 1148)	Q4 (n = 1152)	Q5 (n = 1151)	
Age (years)	50.93 ± 16.61	51.06 ± 17.72	50.92 ± 16.88	51.48 ± 17.31	48.75 ± 16.41	0.001
Gender (n, %)						< 0.001
Male	332 (28.87%)	439 (38.21%)	503 (43.82%)	630 (54.69%)	821 (71.33%)	
Female	818 (71.13%)	710 (61.79%)	645 (56.18%)	522 (45.31%)	330 (28.67%)	
Race/ethnicity (n,%)						< 0.001
Mexican American	115 (10.00%)	144 (12.53%)	142 (12.37%)	128 (11.11%)	137 (11.90%)	
Other Hispanic	136(11.83%)	111 (9.66%)	104 (9.06%)	115 (9.98%)	114 (9.90%)	
Non-Hispanic White	367 (31.91%)	427 (37.16%)	449 (39.11%)	450 (39.06%)	462 (40.14%)	
Non-Hispanic Black	387 (33.65%)	324 (28.20%)	279 (24.30%)	268 (23.26%)	236 (20.50%)	
Non-Hispanic Asian	80 (6.96%)	95 (8.27%)	122(10.63%)	128(11.11%)	147(12.77%)	
Other Race	65 (5.65%)	48 (4.18%)	52 (4.53%)	63 (5.47%)	55 (4.78%)	
Education						< 0.001
< High school graduate	213 (18.52%)	169 (14.71%)	190 (16.55%)	170 (14.76%)	172 (14.94%)	
High school graduate/GED or equivalent	317 (27.57%)	253 (22.02%)	264 (23.00%)	251 (21.79%)	264 (22.94%)	
> High school graduate	620 (53.91%)	727 (63.27%)	694 (60.45%)	731 (63.45%)	715 (62.12%)	
Drinking status (n,%)						0.009
Never	348 (30.26%)	309 (26.89%)	319 (27.79%)	338 (29.34%)	280 (24.33%)	
Moderate consumption	682 (59.30%)	685 (59.62%)	670 (58.36%)	650 (56.42%)	706 (61.34%)	
Excessive consumption	120 (10.43%)	155 (13.49%)	159 (13.85%)	164 (14.24%)	165 (14.34%)	
Smoking status (n,%)						0.009
Yes	490 (42.61%)	438 (38.12%)	477 (41.55%)	480 (41.67%)	525 (45.61%)	
No	660 (57.39%)	711 (61.88%)	671 (58.45%)	672 (58.33%)	626 (54.39%)	
Diabetes status (n,%)	174 (15.13%)	163 (14.19%)	173 (15.07%)	170 (14.76%)	148 (12.86%)	0.639
Hypertension status (n,%)	467(40.61%)	421(36.64%)	444(38.68%)	451(39.15%)	404(35.10%)	0.109
BMI (kg/m^2^)	30.90 ± 7.70	30.37 ± 7.69	30.13 ± 6.81	30.06 ± 7.72	29.99 ± 8.06	0.027
HU (n,%)	245 (21.30%)	208 (18.10%)	217 (18.90%)	221 (19.18%)	196 (17.03%)	< 0.001
Vitamin C intake (mg/day)	33.10 (13.65–68.75)	50.80 (24.45–92.20)	59.85 (29.27–109.03)	66.47 (34.99–118.26)	87.05 (44.45–143.07)	< 0.001
Vitamin B6 intake (mg/day)	1.04 (0.75–1.37)	1.41 (1.11–1.80)	1.69 (1.34–2.12)	1.96 (1.60–2.44)	2.67 (2.10–3.36)	< 0.001
Folate intake (mcg/day)	174.50 (136.50–217.50)	259.50 (217.50–309.50)	323.25 (275.38–380.50)	398.00 (331.00–476.00)	538.00 (436.75–667.00)	< 0.001
Retinol intake(mcg/day)	173.75 (94.62–269.75)	255.00 (158.50–373.50)	314.25 (197.75–459.75)	392.50 (253.38–571.38)	501.50 (323.25–737.25)	< 0.001
Energy intake(kcal/day)	1242.50 (971.56–1526.50)	1640.00 (1373.50–1950.00)	1957.25 (1627.00–2305.62)	2254.25 (1896.38–2684.50)	2779.50 (2333.50–3323.75)	< 0.001
Protein intake(gm/day)	46.48 (34.62–59.99)	62.16 (51.17–75.59)	72.40 (59.45–87.97)	84.39 (70.13–100.85)	105.56 (85.59–129.33)	< 0.001
Carbohydrate intake(gm/day)	136.98 (103.75–175.88)	185.74 (155.10–225.55)	223.85 (184.63–264.59)	260.44 (215.45–314.24)	327.25 (267.43–393.55)	< 0.001
Glucose (mg/dL)	93.00 (86.00–103.00)	93.00 (87.00–101.00)	93.00 (86.00–103.00)	93.00 (87.00–102.00)	93.00 (87.00–101.00)	0.575
Cholesterol (mg/dL)	186.00 (160.00–216.00)	183.00 (159.00–211.00)	185.00 (160.00–212.00)	181.00 (156.00–207.00)	181.00 (156.00–208.00)	< 0.001
TG (mg/dL)	105.00 (79.00–147.00)	107.00 (77.00–158.00)	116.00 (81.00–168.00)	115.00 (81.00–168.00)	119.00 (80.00–179.00)	< 0.001

The prevalence of hyperuricemia was 18.90% (20.15% and 17.79% for males and females, respectively). The results comparing vitamin B1 status and other indicators between hyperuricemia and non-hyperuricemia are shown in Table 2. For men, race, drinking status, BMI, hypertension status, vitamin B1 intake, carbohydrate intake, retinol intake, serum glucose, serum cholesterol and TG level indicators were significantly different between hyperuricemia and non-hyperuricemia. Compared to the participants without hyperuricemia, those with hyperuricemia were more likely to be non-Hispanic white, have hypertension, have higher BMI levels, serum glucose, serum cholesterol, and TG levels, and have lower dietary vitamin B1 intake, carbohydrate intake, retinol intake. In women, patients with hyperuricemia tended to be older, had a higher prevalence of diabetes, hypertension, and higher blood glucose, serum cholesterol, and TG levels, in addition to a lower dietary carbohydrate intake and vitamin C intake (All *P* < 0.05).

**Table 2:** Characteristics of the participants with or without hyperuricemia.

Characteristics	Male		*P*-value	Female		*P*-value
	Non-HU (n = 2176)	HU (n = 549)		Non-HU (n = 2487)	HU (n = 538)	
Age(years)	52.00 (36.00–64.00)	52.00 (36.00–65.00)	0.781	49.00 (35.00–62.00)	60.00 (48.00–69.00)	< 0.001
Race/ethnicity (n,%)			0.003			< 0.001
Mexican American	260 (11.95%)	47 (8.56%)		320 (12.87%)	39 (7.25%)	
Other Hispanic	224 (10.29%)	63 (11.48%)		261 (10.49%)	32 (5.95%)	
Non-Hispanic White	850(39.06%)	190(34.61%)		903 (36.31%)	212(39.41%)	
Non-Hispanic Black	533(24.49%)	138(25.14%)		637 (25.61%)	186(34.57%)	
Non-Hispanic Asian	194 (8.92%)	73 (13.30%)		262 (10.53%)	43 (7.99%)	
Other Race—Including Multi-Racial	115 (5.28%)	38 (6.92%)		104 (4.18%)	26 (4.83%)	
Education level (n, %)			0.252			0.112
< High school graduate	388 (17.83%)	82 (14.94%)		378 (15.20%)	66 (12.27%)	
High school graduate/GED or equivalent	531(24.40%)	134(24.41%)		549(22.07%)	135(25.09%)	
> High school graduate	125(57.77%)	333(60.66%)		1560(62.73%)	337(62.64%)	
Drinking status (n, %)			0.001			0.056
never	559(25.69%)	113(20.58%)		735 (29.55%)	187(34.76%)	
moderate consumption	1245(57.22%)	309(56.28%)		1530(61.52%)	309(57.43%)	
excessive consumption	372 (17.10%)	127(23.13%)		222 (8.93%)	42 (7.81%)	
Smoking status (n, %)			0.503			0.001
Yes	1105 (50.78%)	270 (49.18%)		819 (32.93%)	216 (40.15%)	
No	1071 (49.22%)	279 (50.82%)		1668 (67.07%)	322 (59.85%)	
BMI (kg/m^2^)	28.10 (24.80–32.30)	31.00 (27.30–36.10)	< 0.001	28.60 (24.30–33.60)	34.00 (28.72–40.18)	< 0.001
Diabetes status (n, %)	373 (17.14%)	75 (13.66%)	0.114	263 (10.57%)	117(21.75%)	< 0.001
Hypertension status (n, %)	780 (35.85%)	254 (46.27%)	< 0.001	798 (32.09%)	355 (65.99%)	< 0.001
VitaminB1 intake (mg/day)	1.62 (1.20–2.14)	1.54 (1.11–2.03)	0.015	1.24 (0.92–1.61)	1.22 (0.88–1.58)	0.158
Energy intake(kcal/day)	2239.50 (1723.50–2796.62)	2196.50 (1653.50–2755.25)	0.333	1708.50 (1327.50–2163.75)	1679.00 (1306.00–2081.25)	0.113
Protein intake(gm/day)	84.43 (64.56–108.16)	82.84 (64.77–108.20)	0.570	63.43 (48.95–82.40)	63.58 (50.03–79.08)	0.169
Carbohydrate intake(gm/day)	252.29 (187.64–327.27)	239.15 (179.44–325.13)	0.108	199.86 (153.30–258.44)	190.52 (145.80–248.37)	0.008
Retinol intake(mcg/day)	346.50 (199.88–546.12)	306.50 (177.00–499.50)	< 0.001	288.00 (170.50–446.25)	277.75 (164.12–445.12)	0.227
Vitamin C intake(mg/day)	60.38 (26.60–112.19)	56.15 (26.85–107.45)	0.628	58.00 (27.20–104.15)	52.80 (25.59–93.61)	0.020
Vitamin B6 intake(mg/day)	1.96 (1.43–2.72)	1.95 (1.44–2.73)	0.467	1.49 (1.08–2.04)	1.49 (1.10–1.95)	0.312
Folate intake(mcg/day)	361.50 (268.88–501.50)	353.50 (251.50–487.00)	0.304	291.50 (210.00–392.00)	277.00 (196.00–381.25)	0.091
Glucose (mg/dL)	94.00 (88.00–104.00)	95.00 (89.00–106.00)	0.022	91.00 (85.00–99.00)	96.00 (89.00–110.00)	< 0.001
Cholesterol (mg/dL)	177.00 (152.00–205.00)	187.00 (160.00–220.00)	< 0.001	185.00 (162.00–213.00)	188.00 (160.25–223.00)	0.021
TG (mg/dL)	114.50 (80.00–172.25)	137.00 (98.00–213.00)	< 0.001	102.00 (74.00–146.00)	132.00 (96.00–177.75)	< 0.001

A multivariate model was used to investigate the association between vitamin B1 intake and hyperuricemia, as shown in Table 3. In this cross-sectional study, the association between dietary vitamin B1 intake and hyperuricemia was revealed based on analysis stratified by gender. In the male population, a significant negative association between vitamin B1 intake and HU prevalence was found in models 1, 2 and 3, with linear trend tests showing a statistically significant difference (*P* values are 0.007, 0.028 and 0.028, respectively). In model 3, after controlling for age, race/ethnicity, smoking status, alcohol consumption status, educational background, hypertension status and diabetes status, protein intake, carbohydrate intake, retinol intake, vitamin B6 intake, folate intake, BMI, cholesterol, and TG, an inverse trend was also observed for higher vitamin B1 intake and HU risk. When compared to those consuming less than 0.94 mg Vitamin B1 daily, the relative odds of hyperuricemia were significantly decreased by 0.75 times among those that were consuming 0.94–1.24 mg of vitamin B1 daily (OR = 0.75, 95% CI 0.52, 1.09), 0.70 times among participants who consumed 1.25–1.55 mg daily (OR = 0.70, 95% CI 0.48, 1.02), 0.66 times among those consuming 1.56–1.99 mg daily (OR = 0.66, 95% CI 0.44, 0.99), and by 0.55 times among those consuming 2.00 mg or more daily (OR = 0.55, 95% CI 0.34, 0.90). However, no significant differences were found between dietary vitamin B1 intake and risk of hyperuricemia in women. Among the women, after adjustment for multiple covariates, compared to Q1, the adjusted ORs of HU are 1.05 (95% CI 0.69, 1.60) for Q4 (those consuming 1.56–1.99 mg vitamin B1 daily), 0.75 (95% CI 0.42, 1.34) for Q5 (those consuming 2.00 mg or more), and the *P* for trend is 0.876.

**Table 3:** Adjusted odds ratios of hyperuricemia among participants associated with vitamin B1 intake.

		Dietary vitamin B1 intake (mg/day)					*P* for tend
		Q1 (< 0.94) (n = 1150)	Q2 (0.94–1.24) (n = 1149)	Q3 (1.25–1.55) (n = 1148)	Q4 (1.56–1.99) (n = 1152)	Q5 (> 2.00) (n = 1151)	
Male (n = 2725)	Model I^a^	Reference	0.76 (0.54, 1.06)	0.76 (0.54, 1.06)	0.69 (0.50, 0.95)	0.64 (0.47, 0.87)	0.007
	Model II^b^	Reference	0.74 (0.52, 1.06)	0.72 (0.50, 1.04)	0.68 (0.46, 1.00)	0.57 (0.35, 0.91)	0.028
	Model III^c^	Reference	0.75 (0.52, 1.09)	0.70 (0.48, 1.02)	0.66 (0.44, 0.99)	0.55 (0.34, 0.90)	0.028
Female (n = 3025)	Model I^a^	Reference	0.82 (0.63, 1.08)	0.91 (0.69, 1.20)	0.96 (0.72, 1.28)	0.80 (0.56, 1.16)	0.503
	Model II^b^	Reference	0.89 (0.66, 1.22)	1.00 (0.70, 1.41)	1.10 (0.72, 1.68)	0.78 (0.44, 1.39)	0.989
	Model III^c^	Reference	0.87 (0.64, 1.19)	0.97 (0.68, 1.38)	1.05 (0.69, 1.60)	0.75 (0.42, 1.34)	0.876

^a^Adjusted for age, race/ethnicity;

^b^Adjusted for age, race/ethnicity, hypertension status, BMI, protein intake, carbohydrate intake, vitamin B6 intake, folate intake, and retinol intake;

^c^Adjusted for age, race/ethnicity, smoking status, drinking status, education background, hypertension status, diabetes status, protein intake, carbohydrate intake, retinol intake, vitamin B6 intake, folate intake, BMI, cholesterol and TG.

This study will further stratify the study population to explore the relationship between vitamin B1 and hyperuricemia among subgroups, as shown in Table 4. First, the vitamin B1 five groups Q1-Q5 of Non-Hispanic White were observed in race, and the OR values were 0.79, 0.80, 0.65, 0.43, respectively, and *P* for tend was 0.017. No significant OR values were observed among the other groups.In the Middle age group (42–60 years old), with the increase of vitamin B1 intake (Q1–Q5), the OR values were 0.72, 0.64, 0.64 and 0.44, respectively. The *P* value was 0.023. However, no relationship between vitamin B1 intake and HU was demonstrated in BMI and alcohol consumption status groups.

**Table 4:** Participants' data on vitamin B1 intake were analyzed by stratified subgroups.

Covariates		Dietary vitamin B1 intake (mg/day)					*P* for tend
		Q1 (< 0.94) (n = 1150)	Q2 (0.94–1.24) (n = 1149)	Q3 (1.25–1.55) (n = 1148)	Q4 (1.56–1.99) (n = 1152)	Q5 (> 2.00) (n = 1151)	
Race/ethnicity^a^							
	Mexican American	Reference	0.62 (0.29, 1.33)	0.58 (0.25, 1.38)	1.09 (0.44, 2.68)	0.62 (0.18, 2.08)	0.936
	Other Hispanic	Reference	0.89 (0.41, 1.94)	0.71 (0.31, 1.64)	0.53 (0.21, 1.29)	0.35 (0.11, 1.15)	0.068
	Non-Hispanic White	Reference	0.79 (0.54, 1.17)	0.80 (0.53, 1.20)	0.65 (0.41, 1.04)	0.43 (0.24, 0.80)	0.017
	Non-Hispanic Black	Reference	0.83 (0.55, 1.25)	1.01 (0.64, 1.59)	0.85 (0.49, 1.45)	0.92 (0.46, 1.85)	0.823
	Non-Hispanic Asian	Reference	0.86 (0.36, 2.03)	0.75 (0.31, 1.80)	1.42 (0.56, 3.62)	0.70 (0.23, 2.18)	0.986
	Other Race—Including Multi-Racial	Reference	1.15 (0.39, 3.35)	0.96 (0.32, 2.87)	1.17 (0.37, 3.69)	2.25 (0.55, 9.28)	0.413
Age^b^							
	Low (21–41 years)	Reference	0.69 (0.43, 1.09)	0.88 (0.54, 1.43)	0.90 (0.53, 1.53)	0.77 (0.39, 1.50)	0.814
	Middle (42–60 years)	Reference	0.72 (0.47, 1.11)	0.64 (0.41, 1.00)	0.64 (0.40, 1.05)	0.44 (0.24, 0.83)	0.023
	High (> 60 years)	Reference	0.93 (0.66, 1.31)	0.94 (0.65, 1.38)	0.88 (0.57, 1.36)	0.71 (0.40, 1.29)	0.388
BMI^c^							
	Low (14.6–26.3)	Reference	0.90 (0.54, 1.50)	0.80 (0.45, 1.40)	0.99 (0.54, 1.80)	0.76 (0.36, 1.64)	0.681
	Middle (26.4–32.0)	Reference	0.98 (0.66, 1.46)	1.03 (0.68, 1.57)	0.89 (0.55, 1.44)	0.66 (0.35, 1.25)	0.316
	High (> 32.1)	Reference	0.67 (0.48, 0.93)	0.66 (0.46, 0.93)	0.67 (0.45, 0.99)	0.58 (0.35, 0.97)	0.066
Drinking status^d^							
	Never	Reference	0.93 (0.60, 1.43)	0.65 (0.40, 1.05)	0.82 (0.48, 1.41)	0.67 (0.33, 1.36)	0.244
	Moderate consumption	Reference	0.77 (0.57, 1.04)	0.90 (0.65, 1.24)	0.87 (0.60, 1.25)	0.66 (0.41, 1.05)	0.303
	Excessive consumption	Reference	0.65 (0.35, 1.22)	0.73 (0.38, 1.40)	0.50 (0.24, 1.04)	0.46 (0.18, 1.19)	0.098

^a^Adjusted for age, hypertension status, BMI, protein intake, carbohydrate intake, vitamin B6 intake, folate intake, and retinol intake.

^b^Adjusted for race/ethnicity, hypertension status, BMI, protein intake, carbohydrate intake, vitamin B6 intake, folate intake, and retinol intake.

^c^Adjusted for age, race/ethnicity, hypertension status, protein intake, carbohydrate intake, vitamin B6 intake, folate intake, and retinol intake.

^d^Adjusted for age, race/ethnicity, hypertension status, BMI, protein intake, carbohydrate intake, vitamin B6 intake, folate intake, and retinol intake.

**Figure 1:**
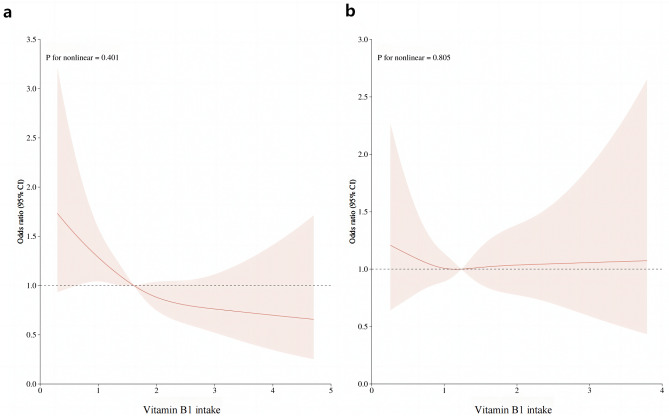
RCS analysis of vitamin B1 intake and risk of hyperuricemia. (**a**) RCS analysis of vitamin B1 in the male population. (**b**) RCS analysis of vitamin B1 in female population. The red line represents the trend, and the light pink area is the 95% confidence interval.

**Figure 2:**
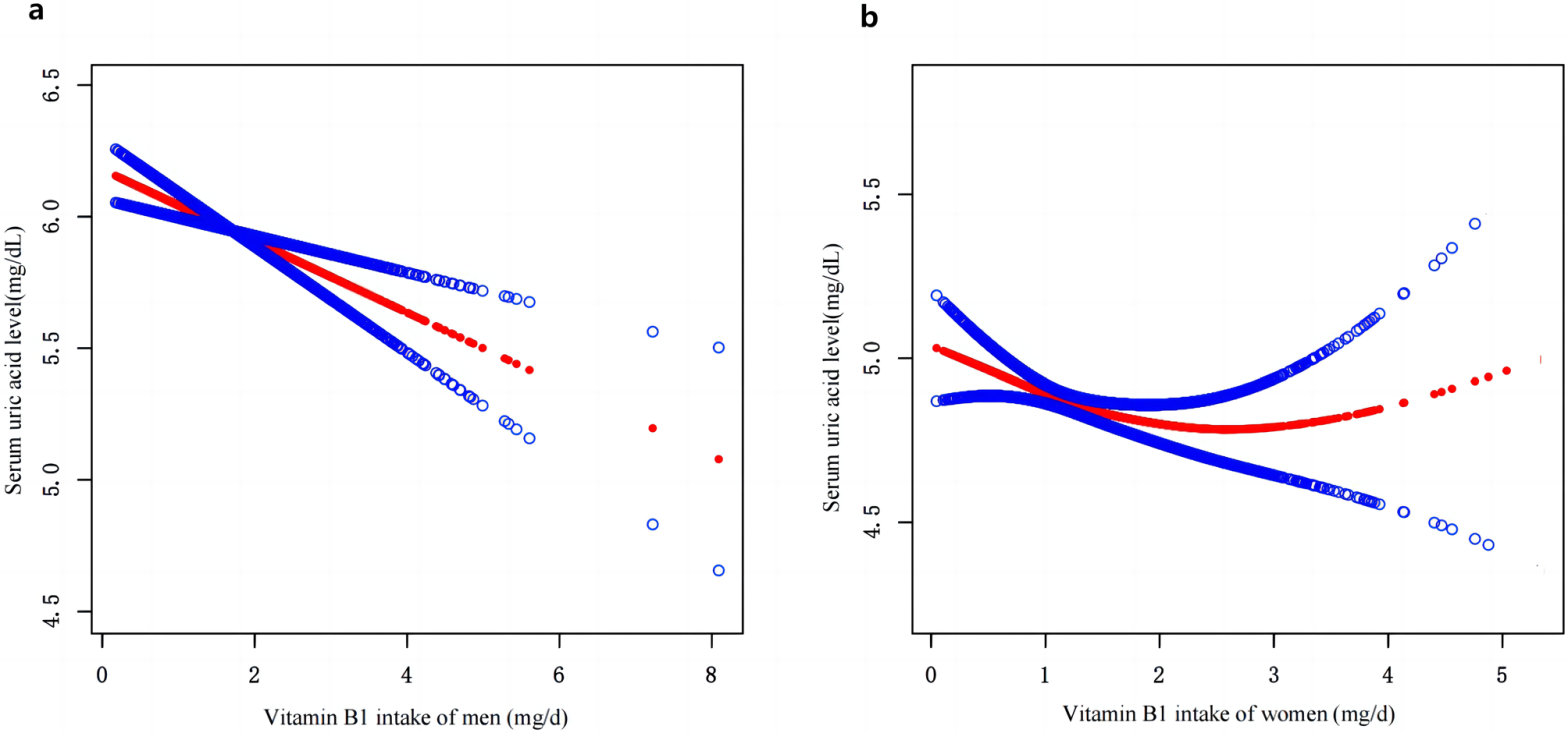
Smooth curve fitting of serum uric acid concentration and dietary vitamin B1 intake of participants.

Following multiple regression, we used the restricted cubic spline curve (RCS) to analyze the dose–response relationship between vitamin B1 and HU. As shown in Fig. 1a, our results indicated a linear relationship between dietary vitamin B1 intake and HU risk in men (*P* = 0.401), with the prevalence of hyperuricemia decreasing with increasing dietary vitamin B1 concentrations. However, this trend was not observed in women (Fig. 1b). In addition, a smoothed curve-fitting analysis was performed to compare the serum uric acid concentration with vitamin B1 intake of the study participants. The results are shown in Fig. 2, which indicates that in men, vitamin B1 intake was inversely related to serum uric acid concentration, meaning that the higher the vitamin B1 intake, the lower the serum uric acid concentration in men. Meanwhile, we plotted no significant relationship between vitamin B1 intake and serum uric acid concentration in women.

## Discussion

In this large population-based study of US adults, we observed a negative association between dietary vitamin B1 intake and HU in men. After adjustment for multiple covariates (age, race/ethnicity, BMI, smoking status, etc.), this association remained significant in men, but no significant association was observed among women.

To the best of our knowledge, this is the first study to show an association between dietary vitamin B1 intake and hyperuricemia in an adult US population. In this study, we used data from nhanes 2017 to 2020 to investigate this association, using a nationally representative sample of US adults, to reveal the association between dietary vitamin B1 intake and hyperuricemia in men and women. A preclinical study has shown the effect of thiamine in reducing uric acid levels in diabetic rats^9^. Thiamin reduces the substrates of the upregulated catabolic pathway involved in uric acid synthesis. One study suggested that treatment with thiamine as a therapeutic agent can alleviate serum uric acid levels in patients with AUD (alcohol use disorder), whether or not they exhibit liver damage. Abstinence and treatment with thiamine could alleviate hyperuricemia in heavy drinkers with mild or no ALD^10^. In addition, benfotiamine which is a derivative of thiamine reduces the effect of uric acid by increasing the serum concentration of nitrite/nitrate at a 4-week low-dose (70 mg/kg/day) regimen^11^. A cross-sectional study in China showed that dietary vitamin B1 income levels were negatively associated with hu prevalence in the male population and not in the female population^8^. This is consistent with our findings. The potential mechanisms between vitamin B1 intake and HU are still largely unknown, but may be related through antioxidant properties and inflammatory mechanisms. Uric acid is an important endogenous antioxidant with physiologically appropriate concentrations that may be required to reduce oxidative stress in cells of the central nervous system. Uric acid provides up to 55% of the free radical scavenging antioxidant capacity of human plasma^12^, making it one of the major antioxidants in the body^13^. In addition, uric acid itself and/or downstream radicals can act as a biologically active proinflammatory factor involved in the production of intracellular oxidants through the ubiquitous NADPH oxidase-dependent pathway, leading to oxidative stress^14^. HU is most often caused by decreased renal uric acid excretion, and excessive serum uric acid levels can lead to precipitation of uric acid crystals in the connective tissue of joints, leading to gouty arthritis and precipitation in the renal tubules, leading to acute tubular necrosis^15^. Thiamine has antioxidative effects. It protects the neutrophil sulfhydryl groups from oxidation by the system^16^ and shows significant inhibition of oxygen radicals produced by neutrophils^17^.Fat soluble form of vitamin B1 could modulate the macrophage inflammatory response against the bacterial toxin-induced inflammation by regulating signaling intermediates such as MAPK/PKC/IΚB leading to the expression of NF-ΚB-dependent inflammatory markers^18^. Therefore, vitamin B1 supplementation could be beneficial in the prevention of inflammation as well as pathological conditions caused by oxidative stress.

Hyperuricemia needs more attention as an early and major cause of gout. Diet has also been hypothesized to be a contributing factor to hyperuricemia, with increased dietary purines leading to increased urate production^19^. Due to the interventional nature of diet, there will be an increasing emphasis on the management of various nutrient intakes in hyperuricemia. A large body of literature focuses on assessing the association between diet and hyperuricemia. For example, red meat, seafood, sugary beverages, alcohol and animal protein have been identified to be associated with an increased risk of hyperuricemia^20^. One study indicated that after adjusting for all covariates, only the vitamin B group had a significant total effect on hyperuricemia^21^. Similarly, our study suggests that increased intake of dietary vitamin B1 from food sources may reduce the risk of HU in men.

There was no negative association between dietary vitamin B1 intake levels and HU prevalence in the female population, which may be related to gender differences in uric acid metabolism. The level of uric acid metabolism is affected by factors such as sex hormones, visceral obesity and muscle mass^22^. Serum urate concentrations on average about 1 mg/dl higher in men than in women in adulthood, but serum uric acid levels in women were significantly higher around the age of natural menopause^23^. In a clinical study of alcohol-related liver disease (ALD), it was noted that uric acid levels were significantly lower in males than in females in hospitalised patients treated with vitamin B1^10^. Furthermore, for men, one study reported that low testosterone levels were associated with elevated uric acid levels. In postmenopausal women, a significant increase in serum uric acid concentration was observed. After hormone replacement therapy, uric acid decreased significantly^24^. The above studies all indicate the effect of gender differences on uric acid levels.

Our study has several highlights. First, we used a large nationally representative sample in the general U.S. population, increasing the reliability of the results. This is the first study to assess the association between vitamin B1 intake and the risk of hyperuricemia in US adults. Second, we adjusted for a number of important confounders. Third, a standardized protocol was followed, and trained staff were used to obtain basic information about the study population. In particular, a standardized assessment instrument was used for dietary nutrient intake to improve the accuracy and validity of the data obtained.

However, our study has some limitations. First, this was a cross-sectional study, limiting the definition of direct causality of vitamin B1 and HU, and further prospective longitudinal studies are important to support the conclusions. Second, the study used 24-h dietary recall to estimate dietary intake levels, which may be subject to recall bias, and also does not accurately describe long-term vitamin B1 intake. Although we controlled for several confounding factors, we cannot get rid of the possibility of residual confusion caused by unmeasured confounding factors.

Our study suggests that that dietary vitamin B1 intake is negatively associated with hyperuricemia in men among US adults. Further-more, large prospective cohort studies need to be performed to support our findings.

## Conclusions

Our study suggests that that dietary vitamin B1 intake is negatively associated with hyperuricemia in men among US adults after adjusting for major confounders. Further-more, large prospective cohort studies need to be performed to support our findings.

## Methods

### Study population

Data from National Health and Nutrition Examination Survey (NHANES). NHANES is a national survey that monitors the health and nutritional status of adults and children across the United States. The survey is unique in that it combines interviews and physical examinations. NHANES is run by the National Center for Health Statistics (NCHS). The data including interviews, physical and laboratory examination can be downloaded from the NHANES website (http://www.cdc.gov/nchs/nhanes.htm). The information collected is used to provide important health statistics. The NHANES protocol was approved by the ethical review board of the National Center for Health Statistics Research, written informed consent was obtained from all subjects. Only publicly available data was used in the analysis, and no ethical approval was needed in this study.

**Figure 3:**
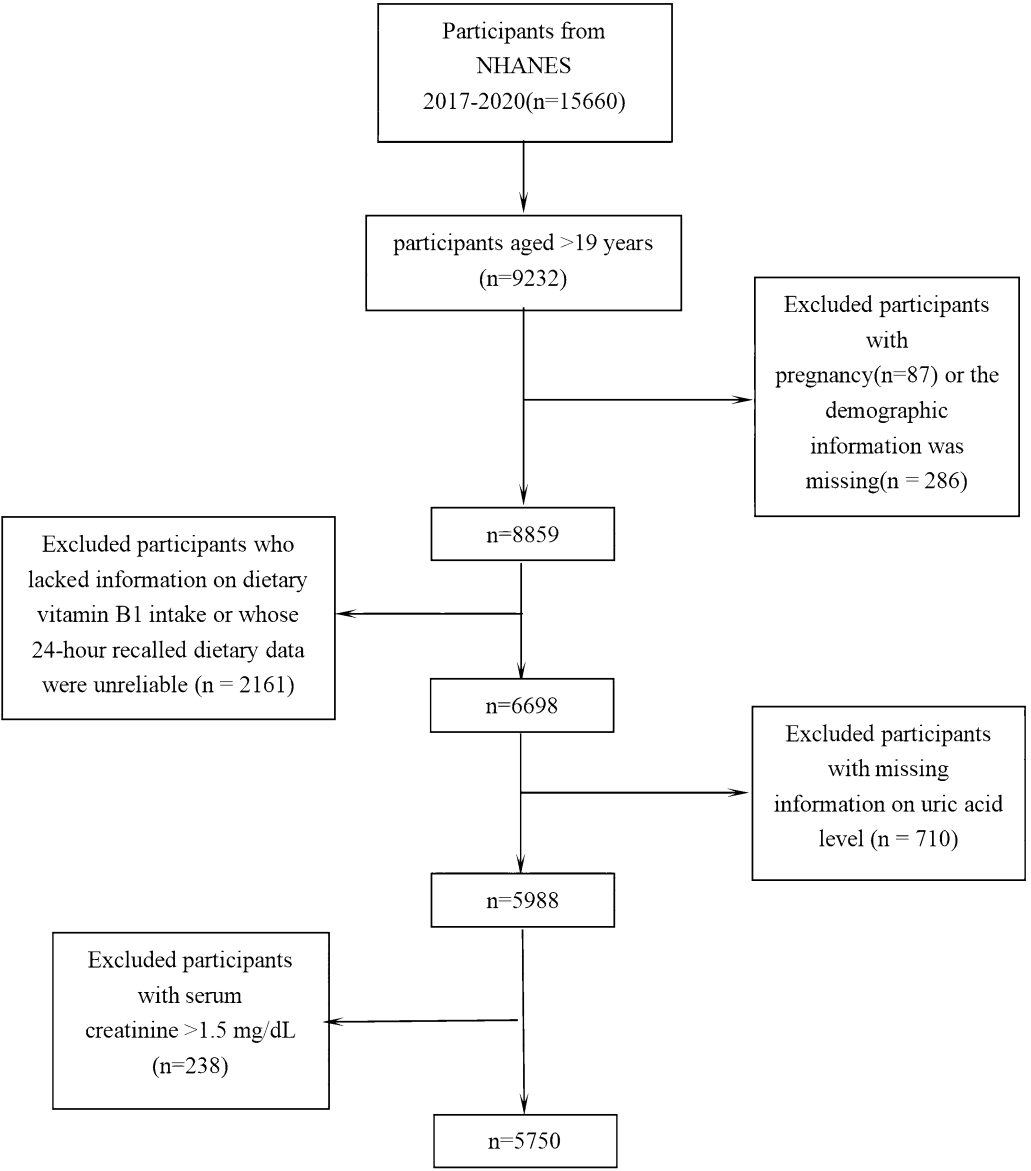
Flow chart of the screening process for the selection of eligible participants.

A total of 9232 participants aged ≥ 20 years were selected from 15,660 participants in the NHANES from March 2017 to March 2020.We excluded pregnant women (n = 87) and participants lacking information or unreliable 24-h recall data on dietary vitamin B1 intake (n = 2161); those participants with missing information on uric acid level (n = 710); and those with serum creatinine > 1.5 mg/dL^25^ were also excluded for considering renal dysfunction(n = 238); and those with missing or incomplete essential information on demographic or total nutrient intakes dietary interviews (n = 286). After exclusion, the total subjects in our study included 5750 adults (2725 men, 3025 women). The filtering process is shown in Fig. 3.

### Measures

The dietary intake of vitamin B1 was assessed using 24-h dietary recall interviews. The dietary intake data were used to estimate the types and amounts of foods and beverages (including all types of water) consumed during the 24-h period prior to the interview (midnight to midnight). Daily aggregates of energy, nutrients, and other food components from foods and beverages were calculated using the US Department of Agriculture Food and Nutrient Database for Dietary Studies (FNDDS 2017–2018 and FNDDS 2019–2020). We used two 24-h dietary recalls to obtain total nutrient intakes. It is important to note that dietary vitamin B1 intake was derived from food intake data and included supplement use. Serum concentrations of UA were measured on a Roche Cobas 6000 Chemistry Analyzer (Roche Diagnostics Corporation, Indianapolis, IN 46,250), where UA is oxidized by allantoin and H_2_O_2_ by the enzyme allantoin. Hyperuricemia was defined as serum uric acid level ≥ 7.0 in men and ≥ 6.0 mg/dl in women^26^.

### Covariates

Sociodemographic characteristics included age, gender, ethnicity (Mexican American, other Hispanic, Non-Hispanic White, Non-Hispanic Black, Non-Hispanic Asian and other race), and educational level. Educational level was divided into less than high school graduate, high school graduate/GED or equivalent and above high school. Other covariates included smoking status (smoking at least 100 cigarettes in life or not), and alcohol consumption (never, moderate consumption and excessive consumption). A history of hypertension or diabetes was defined as a self-reported physician’s diagnosis of hypertension or diabetes.

### Statistical analyses

The continuous variables were described by median and interquartile spacing (skewed distributed data), and categorical variables were presented as percentages. Differences between continuous variables were assessed by Kruskal–Wallis H test. Differences between categorical variables were evaluated by the chi-square test. Multivariate logistic regression analysis was used to estimate the odds ratio (OR) and 95% confidence interval (CI) of HU, according to the vitamin B1 intake quintile for male and female separately, with the lowest quintile being considered as the references, respectively. Covariates were selected based on a number of published studies. Indicators collinearity check was performed using the variance inflation factor (VIF). Model 1 is controlled by age and race/ethnicity. On the basis of model 1, Model 2 was adjusted for age, BMI, hypertension status, protein intake, carbohydrate intake, vitamin B6 intake, folic acid intake, retinol intake and TG. On the basis of model 2, Model 3 was further adjusted for smoking status, education background, diabetes status and cholesterol. On the basis of multiple logistic regression analysis, the linear trend test was conducted for the median intake of dietary vitamin B1. The dose–response relationship was determined by the restricted cubic spline(RCS) method. Smooth curve fitting was used to show the relationship between participants' serum uric acid concentration and vitamin B1 intake.

For all statistical analyses, data were analyzed with the use of the statistical packages R (The R Foundation; http://www.r-project.org; version 4.2.0) and EmpowerStats (www.empowerstats.net, X&Y solutions, Inc. Boston, Massachusetts). A two-sided *P* value < 0.05 was considered to be statistically significant.

## Data Availability

The datasets generated during and/or analysed during the current study are available in the [NHANES] repository, [http://www.cdc.gov/nchs/nhanes.htm].
